# Laser Micromachining in Fabrication of Reverse-µEDM Tools for Producing Arrayed Protrusions

**DOI:** 10.3390/mi13020306

**Published:** 2022-02-17

**Authors:** Hreetabh Kishore, Chandrakant Kumar Nirala, Anupam Agrawal

**Affiliations:** Department of Mechanical Engineering, Indian Institute of Technology Ropar, Rupnagar 140001, Punjab, India; 2017mez0007@iitrpr.ac.in (H.K.); anupam@iitrpr.ac.in (A.A.)

**Keywords:** Reverse-μEDM, Nd: YAG LBµM, micro-holes, burrs, protrusions

## Abstract

This paper focuses on the fabrication of high-quality novel products using a µEDM process variant called Reverse-µEDM. The tool plate required for the Reverse-µEDM is fabricated using Nd: YAG-based laser beam micromachining (LBµM) at the optimized process parameters. The Grey relation analysis technique is used for optimizing LBµM parameters for producing tool plates with arrayed micro-holes in elliptical and droplet profiles. Titanium sheets of 0.5 mm thickness were used for such micro-holes, which can be used as a Reverse-µEDM tool. The duty cycle (a combination of pulse width and frequency) and current percentage are considered as significant input process parameters for the LBµM affecting the quality of the micro-holes. A duty cycle of 1.25% and a current of 20% were found to be an optimal setting for the fabrication of burr-free shallow striation micro-holes with a minimal dimensional error. Thereafter, analogous protrusions of high dimensional accuracy and minimum deterioration were produced by Reverse-µEDM using the LBµM fabricated tool plates.

## 1. Introduction

The technology for fabricating micro-scale engineering components and features is of interest to the biomedical, optical, and electronical industries [1]. One of the essential components is an arrayed protrusion (or micro pin-fins) of complex cross-sectional profiles (i.e., elliptical, circular, and diamond shapes), which is used for high heat dissipation from high-performance microelectronics [2]. The fabrication of such protrusions requires dedicated technology to achieve high dimensional accuracy and cost-effectiveness in the products. Amongst the recently developed non-conventional micro-fabrication technologies, the Reverse-μEDM process has emerged as a promising technique for fabricating micro-scale protrusions of high aspect ratio [3] and complex cross-sections [4]. Reverse-μEDM works on reversing the conventional µEDM polarity in which material removal takes place due to electron work function and electrical resistivity associated with the discharge energy ratio [5]. These fabricated protrusions have cross-sectional profiles similar to the shape of the micro-holes on the tool plate fabricated by LBμM. Notably, while fabricating a dense array of unconventional protrusions using the above-said process, issues of high machining time and damaged tips of the arrayed structures, particularly in the central zone, are found [6]. Of these two significant issues, the former is related to the process capability. The latter, which is of particular interest to the present research, is due to the poor quality of those micro-holes in the tool plate. Kishore et al. [7] explored LBμM as an auxiliary process used for fabricating tool plates with the desired shape micro-holes in different arrangements. Both the processes were facilitated on a single CNC machine tool with an axial resolution of 0.1 µm. Nevertheless, there remains a presence of cleavage burrs on the side walls of each micro-hole that lead to damaging the subsequently fabricated protrusions. The reason was anticipated to be a poor selection of process parameters.

However, LBµM can machine a wide range of difficult-to-cut materials, such as ceramics [8,9], polymers [10,11], and various metals such as aluminum alloys [12], stainless steel [13], and titanium alloys [14]. The inherent burrs and recast layer formation, especially in metallic samples, results in inaccurate dimensions and poor surface quality of fabricated microcavities. It may be due to the high heat input of an intense laser beam, which directly vaporizes the molten metal from the localized zone, resulting in the vapor and plasma pressure generation from the microcavity [15]. Appropriate heat input parameters can control the excessive burrs and recast layer formation. This includes wavelength [16,17], pulse duration [18,19], and laser power [20], which are the primary LBμM parameters that affect the machining characteristics. Several works were carried out to analyze the effect of these process parameters on microcavity fabrication. Tunna et al. [21] investigated the impact of varying wavelengths (355 nm, 532 nm, and 1064 nm) and laser intensity (0.5–57.9 GWm^−2^) in pulsed Nd: YAG LBμM over the copper foil. They observed the maximum etch per pulse at 532 nm wavelength while the minimum was at 1064 nm due to the higher reflectivity of copper. Leitz et al. [22] conducted a detailed comparative study of pulse durations in micro, nano, pico and femtosecond LBμM. They are followed by Liu et al. [23], who reported that ultra-short laser pulses result in comparatively better precision in micromachining in terms of surface quality but with poor machining responses.

The enhanced machinability of the Nd: YAG-pulsed laser has attracted researchers to investigate other associated process parameters, such as laser intensity, frequency, scanning speed, and line spacing on the *MRR* and surface roughness of thin sheet [24]. Demir et al. [19] investigated the pulse width (12 ns and 200 ns) effect of nanosecond pulsed LBμM on the higher productivity of TiN coatings.

Considering the importance and capability of LBμM, the present work optimizes Nd: YAG-based pulsed fibre LBµM parameters for fabricating high-quality tool plates for the Reverse-μEDM process. The quality is considered in terms of burrs, striation marks, and dimensional accuracy of the machined arrayed micro-holes. The tool plate is then demonstrated for producing damage-free and dimensionally accurate arrayed elliptical and droplet protrusions using the Reverse-μEDM process and thus can be considered the main contribution of the present work.

## 2. Materials and Methods

### 2.1. Complete Process Configuration

**Figure 1:**
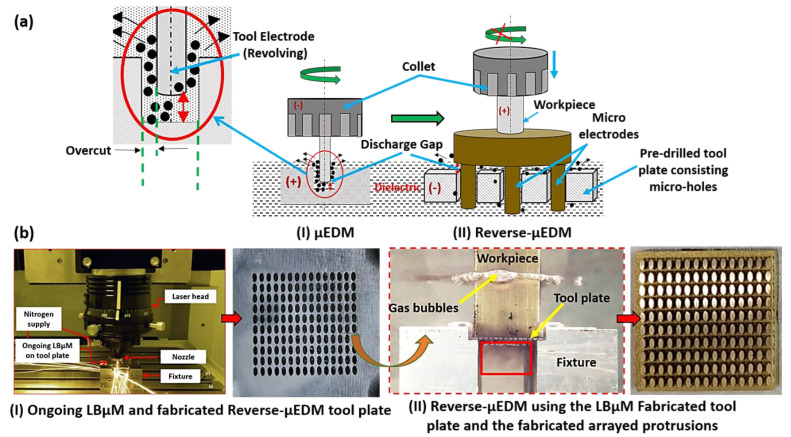
(**a**) Schematic representation of the Reverse-µEDM achieved through µEDM drilling, and (**b**) (I) the LBµM for producing tool plate, and (II) the Reverse-µEDM for fabricating arrayed protrusions.

Schematic for integration of Reverse-µEDM and LBµM for fabrication of the arrayed protrusions as the final product is shown in Figure 1. The Reverse-μEDM process consists of an RC discharge circuit with multiple options for capacitance in parallel connections and discharge voltages, a tool electrode, a workpiece electrode, and a dielectric medium. The stored energy from the capacitor is released instantaneously, due to which electro-thermal erosion occurs from the tool and the workpiece leading to material removal.

Here, the Reverse-µEDM is mainly used to fabricate single or arrayed 3D protruded structures of varying aspect ratios and cross-sectional profiles [25]. The basic tool-work configuration and polarity alteration of µEDM to achieve Reverse-µEDM is depicted in Figure 1a. In Reverse-µEDM, generally, a work material (anode) with a flat face is attached to the non-rotating spindle fed towards the tool plate (cathode). Figure 1b shows that the tool plate has an array of micro-holes in a pattern similar to the required array of protruded structures. These micro-holes are fabricated by progressive LBµM head, customized to gain an identical positional accuracy. Thus, it is possible to fabricate precise micro-holes using LBµM in any profile, which is almost impossible through any other mechanical micromachining.

### 2.2. LBµM Experiments for Fabrication of Reverse-µEDM Tool Plate

**Figure 2:**
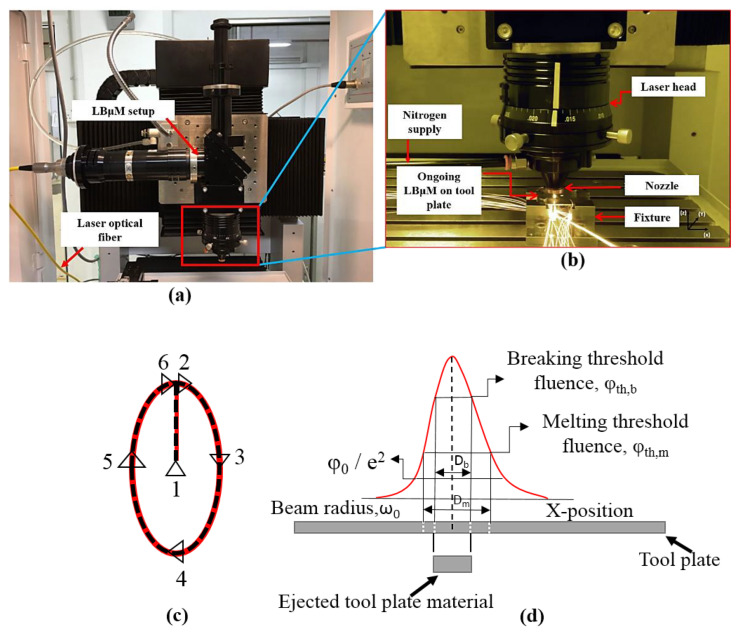
(**a**) The LBμM setup, (**b**) Ongoing LBμM for fabrication of micro-holes, (**c**) trepanning scanning pattern, and (**d**) Gaussian beam profile distribution.

The experiments are performed on a commercially available pure titanium (ASTM Grade 2) sheet of 0.5 mm thickness. The sheet thickness is chosen considering enough margin after the possible erosion while used as a tool plate in the Reverse-µEDM. At the same time, it is also ensured that the available LBµM can cut such a difficult-to-cut material of 0.5 mm thickness. Titanium was chosen for its outstanding high strength, low weight, and corrosion resistance properties. It has led to a diversified range of fruitful applications in MEMS devices fabrication [14]. The nanosecond pulsed Nd: YAG fibre laser (Class 4, iPG Photonics, Yokohama, Japan) is used to fabricate micro-holes of desired shapes (refer to Figure 2a). Pulse width, pulse frequency, and average laser power expressed as the percentage of the applied current to the diode lasers are the essential process parameters considered for optimization study.

Average peak power, resulting in material removal, is an essential parameter for achieving dimensionally accurate high-quality micro-holes is presented through Equations (1)–(3) [20];

*T_p_* = *Duty cycle*/*f* (1)

*P_peak_* = *P_avg_*/*f* × *T_p_* (2)

*E_pulse_* = *P_peak_* × *T_p_* (3)

Here (“*T_p_*”) is pulse width and (“*f*”) is the pulse frequency. It can be observed from Equation (2) that the peak power of the laser is a function of average power (“*P_avg_*”) developed for a given pulse frequency and pulse width. The present work utilizes a smaller spot size (with a minimum spot diameter of 50 μm) high-intensity laser beam profile, usually based on the fluence profile of the Gaussian distribution function with two thresholds beam diameter, as shown in Figure 2d [26]. The irradiated laser beam follows the trepanning scanning tool path (refer to Figure 2c) in the x-axis on the sheet.

**Figure 3:**
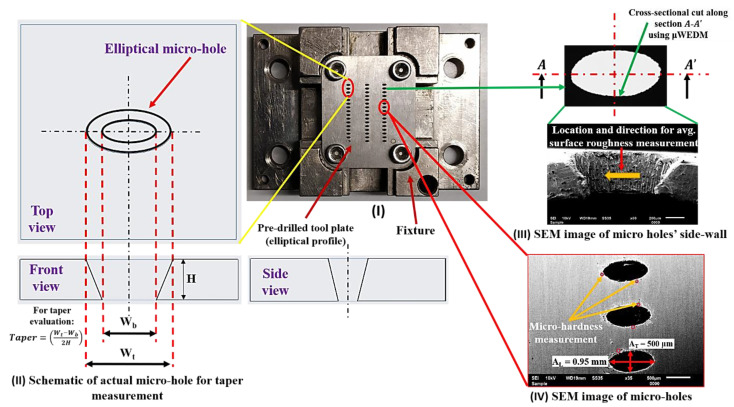
Illustration for estimating (**I**), (**II**) taper, (**III**) *Ra_T_*, and (**IV**) micro-hardness of tool plate.

A dedicated fixture is used to hold the sheet (tool plate for Reverse-µEDM) in such a way that there remains a gap for the free flow of ejected melted material (refer to Figure 3I). The flow of the molten metal is assisted by a shielding gas (nitrogen), which is coaxially supplied to the laser head. An illustration of ongoing LBµM for micro-hole fabrication on the sheet is shown in Figure 2b. Elliptical micro-holes, with the major and minor axes of 950 μm and 500 μm are fabricated at different LBµM parameters. The different LBµM process parameters and their levels considered to perform the experimental runs are summarized in Table 1. A design of experiments (DOE) approach based on Taguchi *L16* orthogonal array is applied to identify the best possible parametric combination with minimum experimental runs required, as shown in Table 2. The auxiliary parameters viz. stand-off distance, assisting gas pressure, and scanning speed is kept constant at 300 µm, 9 bar, and 150 mm/min. The quality criterion of LBµM includes the measured machining responses of each micro-hole in terms of minimum recast layer height, *Ra_T_*, taper, and maximum *MRR_T_*, along with HAZ microhardness. It is noteworthy that each experiment is repeated thrice, and the average response values are tabulated.

The optimal LBµM machining parameters are obtained based on the interaction of multiple responses using Grey relational analysis. The optimal parametric combination is obtained from the highest grey relational grades obtained by calculating the mean of all the coefficients associated with the recorded experimental responses [27]. Moreover, a confirmatory experimental run is conducted to analyze the deviation in the recorded responses, and the discussion in detail on the recorded responses is presented in Section 3.1.

The machined micro-holes in the elliptical cross-section are evaluated for detailed dimensional analysis, chemical composition, and surface characterization alteration. A 2D image acquisition of the fabricated holes is conducted using an optical microscope (ZEISS Axio Vert. A1, Carl Zeiss Microscopy GmbH, Jena, Germany). It is followed by microstructural and EDS analysis on the micro-holes surface using SEM (JSM6610LV, Jeol Ltd. Freising, Germany) equipped with an XFlash 6130 QUANTAX (Bruker Ltd., Bremen, Germany) EDS system. Besides these, various essential LBµM responses were evaluated, such as *MRR_T_*, *Ra_T_* (using roughness tester, Mitutoyo, 178–923E SJ210 Series (Mitutoyo South Asia Pvt. Ltd., New Delhi, India) (refer to Figure 3III). The top and bottom kerf width (“*W_t_*”) and (“*W_b_*”), and taper (refer to Figure 3II) are also evaluated for each micro-hole. For the precise calculation of mass loss, an electronic micro-weighing balance (MYA 21.4Y Microbalance, RADWAG Balances and Scales Ltd., Radom, Poland) with a repeatability of 0.1 µg is used. Another response, i.e., HAZ micro-hardness measurements, is performed using a Vickers hardness testing machine (Wilson Instruments, 402 MVD, Esslingen, Germany) near the cut edge of each micro-hole (refer to Figure 3IV).

### 2.3. Reverse-µEDM Experiments for Fabricated Arrayed Protrusions

**Figure 4:**
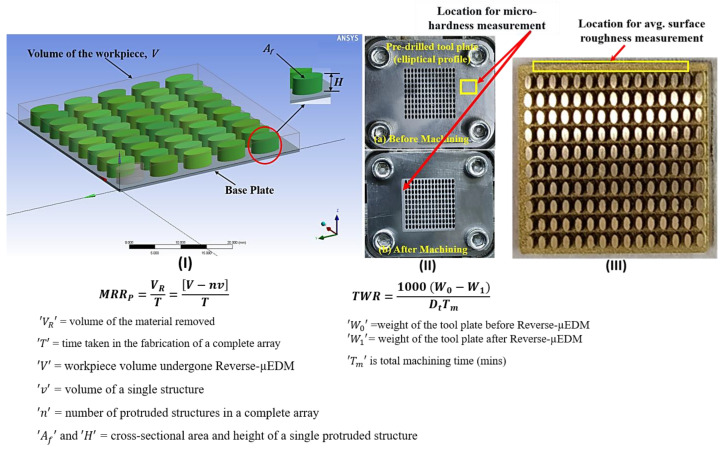
Illustration for (**I**) MRR, (**II**) *TWR* estimation, and (**III**) location for *Ra_P_* measurement.

The obtained optimal LBμM parametric combination is used further to fabricate tool plate consisting of a single or an array of micro-holes as an essential component in Reverse-μEDM for producing arrayed protrusions. The machining conditions for Reverse-μEDM and optimal LBμM are given in Table 3. Three essential output responses, i.e., *MRR_P_*, *Ra_P_*, *TWR*, were rigorously monitored and recorded (approaches are depicted in Figure 4) as they are significantly affected by the nature of the machining environment. In addition to geometrical analysis, surface characterization, “*Ra_P_*” and micro-hardness of tool plates were also evaluated.

## 3. Results and Discussion

This section analyzes the recorded responses mentioned in Section 2 and the optimal parametric combination of LBμM parameters. It is followed by a detailed discussion regarding the dimensional and surface quality evaluations of Reverse-μEDM fabricated arrayed protrusions using tool plates fabricated at optimal LBμM parameters in Section 3.2.

### 3.1. LBµM Experimental Results

**Figure 5:**
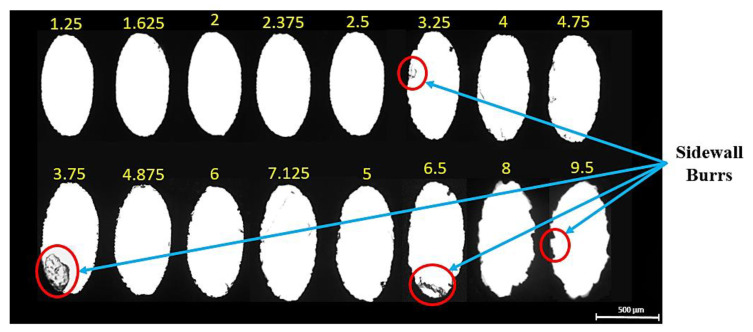
Micro-holes at different parametric combinations (indicated by corresponding duty cycle values).

**Figure 6:**
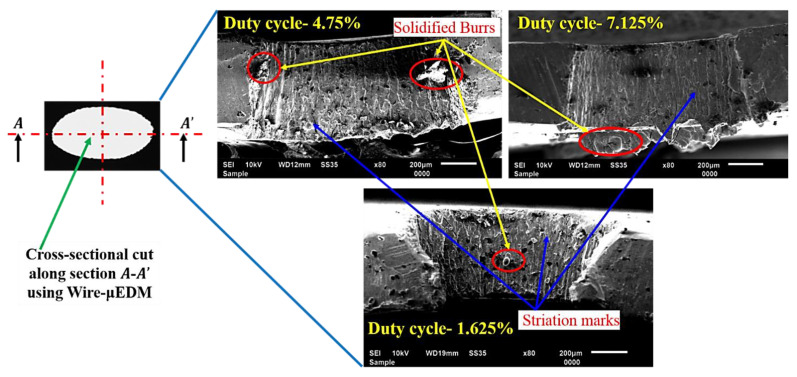
SEM images of holes’ cut-section depicting striation marks.

The optical images of all the fabricated micro-holes at different duty cycles are shown in Figure 5. It is observed that the micro-holes fabricated at lower duty cycles have shown good dimensional accuracy with minimal burr formation than the micro-holes fabricated at higher duty cycles. It may be due to the better efficiency of molten metal removal from the cut kerf at lower duty cycles. Post examination of fabricated micro-holes reveals the striation patterns on the holes’ side wall at lower moderate and higher duty cycles, as shown in Figure 6. It is a well-accepted fact in LBµM that the formation of either uniform or non-uniform striation patterns on the holes’ side walls is solely associated with the molten metal viscosity and purging of assisting gas. The purging gas enhances the cooling effect and generates the drag force on the molten metal through the cut kerf, which may stick to the micro-holes’ side walls. The rate of flow of molten metal (fluid strain) also changes due to the movement of the nozzle head along with the desired holes’ profile, which alters the amount of molten metal purging outside the cut kerf of the micro-holes. The lesser molten metal’s viscosity and surface tension along the tool plate thickness results in the retardation of the molten metal streamlines to accumulate at the bottom of the micro-hole. This results in better dimensional accuracy and a shallow striation pattern at lower duty cycles than the ejected melted metal at higher duty cycles, shown in Figure 6 (recorded data are tabulated in Table 2).

#### 3.1.1. Microstructure and EDS

**Figure 7:**
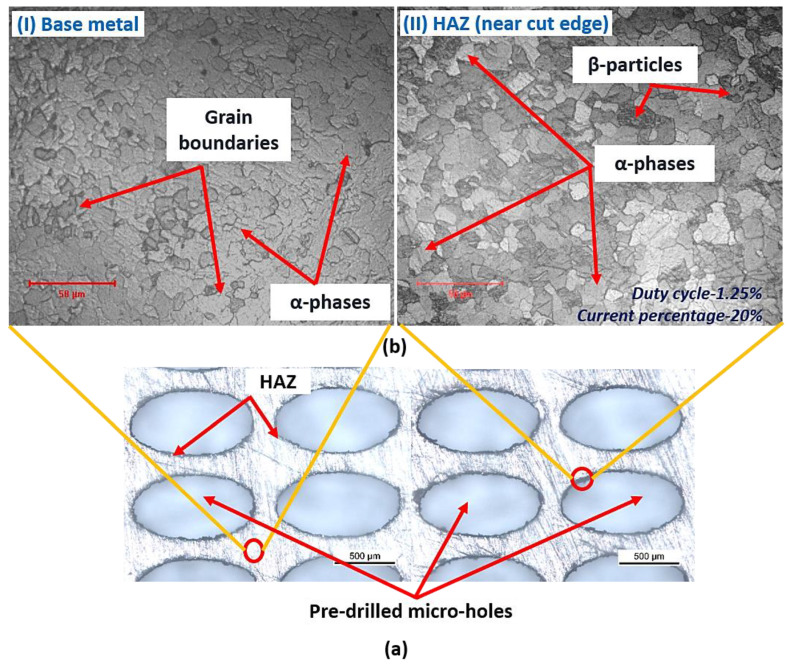
(**a**) Tool plate with micro-holes, and (**b**) microstructure at (**I**) base metal surface, and (**II**) at HAZ location.

During the LBμM process, the thermal gradient is generated near the cut edge as the machining progresses. The developed thermal gradient alters the microstructure of the base metal, as observed in a small zone near the cut edge as HAZ. Figure 7b(I,II) shows the base metal’s microstructure and HAZ developed at a duty cycle of 1.25% and a current percentage of 20%. Careful observation of the HAZ microstructure also shows the difference in the microstructural details compared to the base metal. The grains are uniformly distributed in the HAZ region compared to the random distribution on the bulk metal surface at lower duty cycles. Additionally, the amount may be altered due to the wide range of process parameters and thermal strains generated at high-intensity laser beam irradiation.

**Figure 8:**
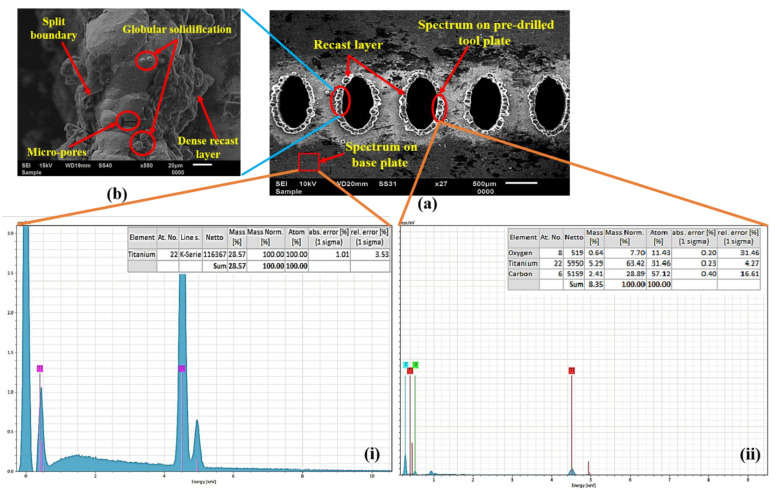
(**a**) EDS plots on tool plate (i) base metal, (ii) cut edge of micro-hole, and (**b**) enlarge view of the cut edge.

Additionally, the extent of the alteration in the HAZ region is also analyzed through the elemental distribution of the titanium tool plate. Quantitatively, the elemental data were presented to better compare the base metal and the HAZ region of the micro-hole shown in Figure 8a(i,ii). To illustrate both regions on the tool plate surface, the spectrum is highlighted with a red box in the corresponding SEM image of the micro-holes shown in Figure 8a.

A significant alteration is found in the elemental composition near the cut edge, presenting the elements’ details from the EDS plot. The presence of smaller oxidation zones and carbide formation may be due to the reaction of the molten metal to the environmental gases present in the machining chamber or surface oxidation. However, the EDS of the base metal shows only the titanium content. Some micro-sized split boundaries, micropores, and globular solidification impressions are found at the cut edges, as shown in Figure 8b. These features might be occurring due to sudden vaporization and bubble formation in the melted metal layer. A dense recast layer adjacent to the micro-holes cut edge is evidenced, of which sufficient explanation is provided in the following sub-section.

#### 3.1.2. Recast Layer and *MRR*

The top surface of the workpiece is subjected to focused laser beam irradiation results in sequential heating, melting, and vaporization, leading to the metal’s removal through micro-holes. While some portion of the resolidified molten metal gets interlocked at the cut edge, known as the recast layer. The SEM image of micro-holes confirms the recast layer formation, shown in Figure 8a,b.

**Figure 9:**
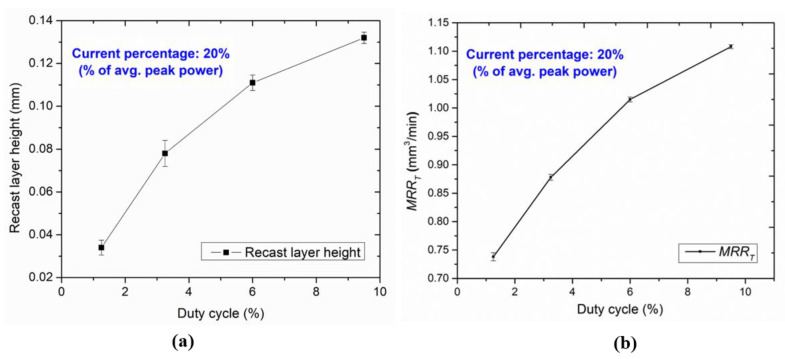
Variation of (**a**) recast layer heights (**b**) *MRR_T_* with duty cycles.

Figure 9a shows the line graph of the recast layer height against the increasing duty cycle at a constant current of 20% (% of avg. peak power) for all micro-holes. It is observed that the trend for recast layer height is growing with an increase when the duty cycle and current percentage lie from 20–80% and between 0.034 and 0.132 mm. This is because of the longer interaction time between the tool plate and the intense laser beam at a particular parametric combination. The increasing laser beam energy per unit area, leads to more melting, causing more recast layer formation at the cut edges. Additionally, the high vapor pressure generation on the molten metal surface causes a thermal gradient effect from the adjacent base metal. It generates hot plasma at the top of the cut edge, absorbs laser energy coming towards the base metal surface, for which the intensity of the laser beam is somewhat reduced. At higher duty cycles, high thermal gradients occur between the base metal and cut edge of the tool plate surfaces at a particularly assisting gas pressure resulting in the thicker recast layer height.

The influence of LBμM parameters on *MRR_T_* is also evaluated for the productivity of the micro-holes. The corresponding *MRR_T_* data for each experimental run shown in Table 2 varies from 0.738 to 1.108 mm^3^/min for lower to higher duty cycles. Figure 9b shows the variation for *MRR_T_* at a constant current of 20% (% of avg. peak power). A similar trend was observed for the increased *MRR_T_* with an increasing current percentage from 20% to 80%. With the increasing current percentage (at the higher duty cycle), the tool plate’s top surface is susceptible to intense laser irradiation and faster melting and vaporization, which leads to an improved *MRR_T_*.

#### 3.1.3. Taper

**Figure 10:**
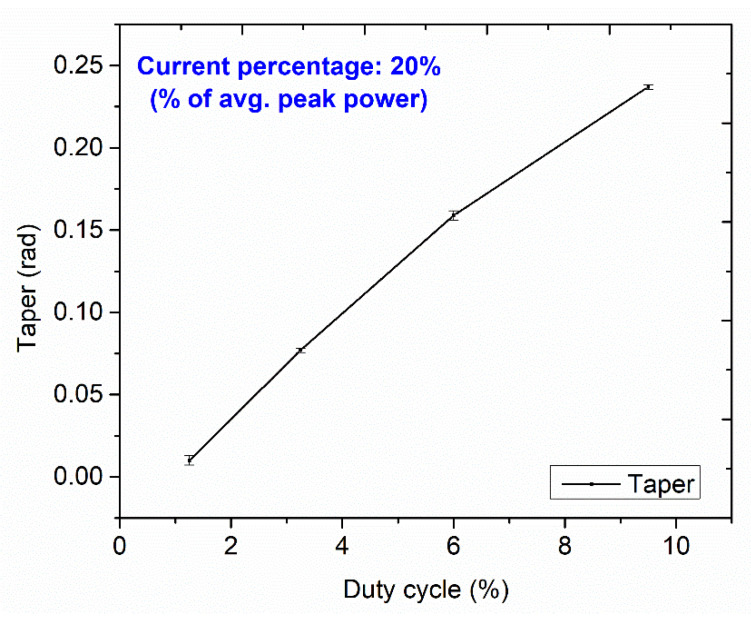
Variation of taper with duty cycles.

The micro-holes cut edge and the side wall surface gets slightly modified, causing the non-identical entrance and exit, producing a taper due to the varying heat input and assisting gas pressure. Figure 10 exhibits the effect of increasing the duty cycle at a constant current percentage of 20% (% of avg. peak power) on the taper of the micro-holes. A similar trend of the increasing taper is observed for every consistent current percentage from 20% to 80%, showing the dominant contribution among other input parameters. As the power of the laser beam increases, the thermal energy of the incident beams directly transfers to the top surface of the tool plate. As a result, the top surface of the tool plate gets severely melted and vaporized and the strength of the beam decreases at the bottom edge, causing metal removal through a narrow gap. In addition, assisting gas pressure causes an increment in the taper by simultaneously cooling and ejecting the melted metal from the top surface of the tool plate at higher duty cycles.

#### 3.1.4. Avg. Surface Roughness, *Ra_T_*

**Figure 11:**
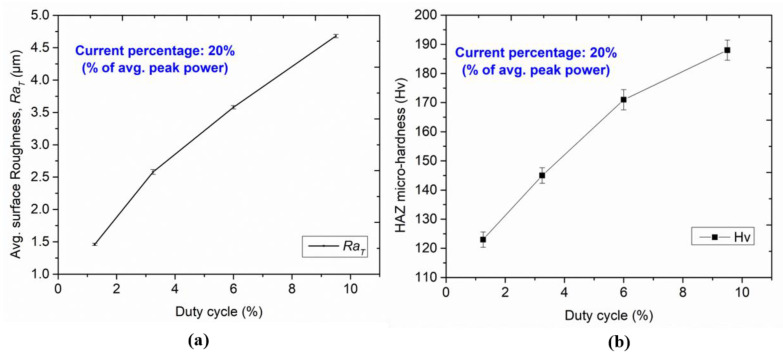
Variation of (**a**) *Ra_T_*, and (**b**) HAZ micro-hardness with duty cycles.

An increase in laser intensity irradiation on the tool plate surface results in higher amounts of molten metal removal. Additionally, the drag force generated by assisting gas pressure purging out the molten metal faster causes unevenness on the side-wall surface of micro-holes. The effect of LBµM parameters on varying *Ra_T_* of the micro-holes’ side wall can be analyzed through the line plot in Figure 11a. The variation of *Ra_T_* value with duty cycle at different current percentages ranging from 20% to 80% is observed as 1.46 to 4.68 µm (refer to Table 2).

It is observed that the *Ra_T_* values are minimal at the lower duty cycle in LBµM. A lower duty cycle ensures the availability of great timing for the laser beam to melt and vaporize the molten metal. Additionally, the melted metal removal from the cut kerf is supported by suitable assisting gas pressure, which drags the molten metal uniformly. However, with an increase in duty cycle, the *Ra_T_* values show an increasing trend. Excessive melt pool formation caused by partial melting of base metal near the cut edge creates non-uniformity on the side wall surface as non-uniform striation marks and high *Ra_T_* values.

#### 3.1.5. Micro-Hardness of HAZ

The variation of HAZ micro-hardness with increasing duty cycle at constant current percentage is illustrated in Figure 11b. A similar trend was observed for increased micro-hardness with an increasing current percentage from 20% to 80%. It is observed that the hardness near the cut edge of each micro-hole is significantly higher than the hardness of the base metal. The formation of intense layers of titanium carbides near the cut edges and varying thermal loading leads to a surface modification at the edges. The formation of these layers may be due to the chosen assisting gas pressure and grain refinement. The traces of carbide formation at the cut edge of the micro-holes are confirmed by EDS analysis, as shown in Figure 8a(ii).

**Figure 12:**
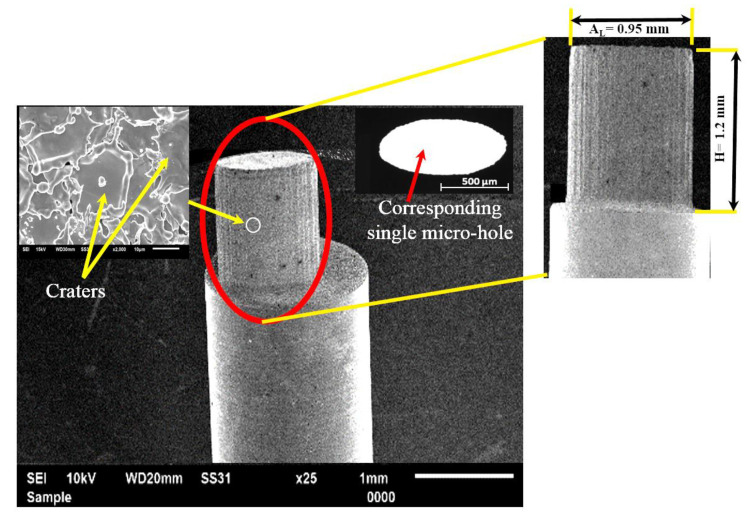
SEM image of single elliptical protrusion produced using micro-hole shown in the inset.

From the optimization, the parametric combination for the first experimental run (refer to Table 2) has shown the best performance for the multi-objective optimization (maximizing *MRR_T_*, HAZ micro-hardness, and minimizing recast layer height, taper, *Ra_T_*). The reproduced machined micro-hole fabricated at the optimal parametric combination (refer to Table 2) has presented satisfactory machining responses with less than 1% error. This is confirmed through an experimental validation run shown in the inset (refer to Figure 12) that has no evidence of side wall burrs and no significant deviation in the taper. This parametric combination is further utilized in the micromachining of single and arrayed micro-holes for producing arrayed protrusions.

### 3.2. Reverse-μEDM Using Optimal Tool Plate

The micromachined single micro-hole at the optimized LBμM parameters is used to fabricate a single protrusion using the Reverse-μEDM. The SEM images of the single elliptical protrusion and the fabricated micro-hole are shown in Figure 12. The fabricated protrusion is almost free from tip damage around its periphery and perpendicular along the orthogonal length.

**Figure 13:**
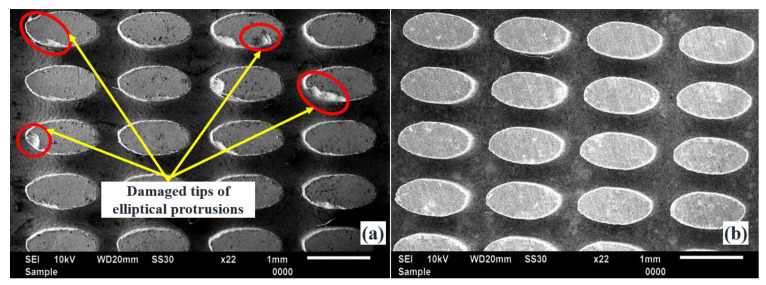
SEM images of fabricated elliptical arrayed protrusions with (**a**) randomly selected and (**b**) optimal LBμM parametric combinations.

The potentiality of the obtained optimal LBμM parametric combination is further used for fabricating an array of elliptical and droplet micro-holes and the subsequent arrayed protrusions. For this, a fabricated tool plate with a collection of 10 × 15 micro-holes is fabricated at two different LBμM conditions: (I) a randomly selected combination (includes a pulse width of 0.75 ms, pulse frequency of 75 Hz, and a current of 20% (% of avg. peak power)), and (II) an optimal parametric combination, as highlighted in Table 2. SEM images of fabricated protruded structures at both mentioned conditions are shown in Figure 13a,b. The fabricated arrayed protrusions, with the tool plate micromachined at the random parametric set, encounter the issue of damaged tips for a few of the structures, as seen in Figure 13a. However, the presence of burrs causes longer machining time due to non-contributing discharge pulses resulting in non-uniform material removal from the workpiece electrode until it gets removed from the entire micro-holes. In contrast, it was not significant while fabricated at optimized LBμM parameters, as shown in Figure 13b. The reason could be well understood by looking back at the micro-hole fabricated at the optimized LBμM parameters, as shown in Figure 12 (inset). Since it has no apparent cleavage or burrs at the micro-hole cut edges, it allows faster machining by restricting high-order discharges and short-circuiting during Reverse-μEDM.

**Figure 14:**
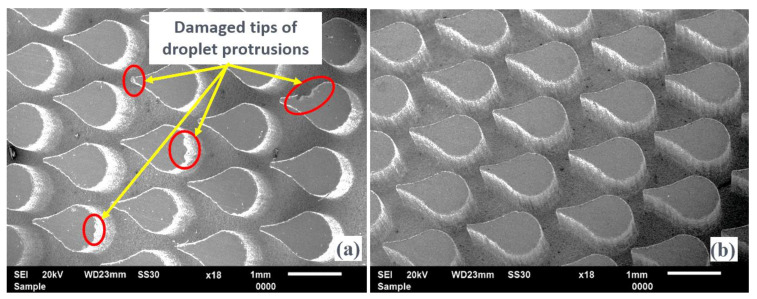
SEM images of fabricated droplet arrayed protrusions with (**a**) randomly selected and (**b**) optimal LBμM parametric combinations.

Moreover, a droplet cross-section profile with a minimum inter-electrode gap of 100 µm in a staggered configuration is also fabricated. Figure 14a,b show partial magnified images of a few protrusions from an array taken at the center.

The recorded dimensions of fabricated protrusions are depicted in the same Figure in which an almost negligible taper is evidenced. The reason for high-quality droplet protrusions adheres to a similar explanation, as reported in the case of arrayed elliptical protrusions.

**Figure 15:**
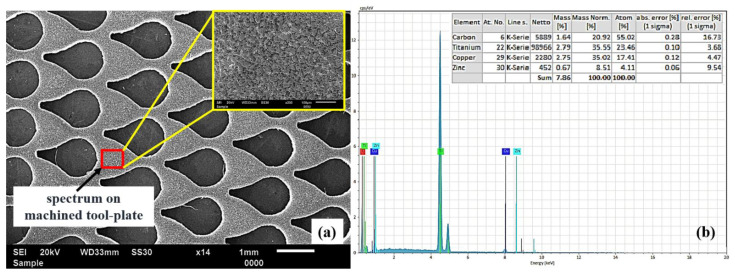
Energy spectrum on (**a**) tool plate after Reverse-µEDM, and (**b**) corresponding EDS plot.

In Reverse-μEDM, the workpiece being as anode and tool plate as cathode, are subjected to generating a new surface due to frequent electrical discharging between them. The alteration in the modified surface of the tool plate is confirmed through elemental analysis captured at the zone where the machining takes place after Reverse-μEDM. Figure 15a depicts the spectrum, whereas Figure 15b shows the various elemental composition percentages on the machined tool plate.

Responses recorded from the Reverse-μEDM are presented in Table 3. *MRR_P_*, TWR and *Ra_P_* recorded improvements of more than 16%, 20%, and 10%, respectively, while using the tool plate fabricated at optimized LBμM parameters. The burr-free tool plate at the optimized LBμM parameter leads to uninterrupted machining, hence, improved *MRR_P_*, *TWR*, and *Ra_P_.* Additionally, it leads to the freezing of abnormal discharges by proper debris evacuation from the discharge gap. However, abnormal discharges due to debris accumulation are significant for increased tool wear in μEDM [28]. As a result, there is less possibility for debris re-solidification, due to its rapid cooling, on the machined tool plate surface in Reverse-μEDM. In contrast, the tool plate fabricated at the optimized LBμM parameters, comparatively, provides more scope for its rapid cooling during Reverse-μEDM. Hence, it leads to slightly better micro-hardness of the pre-drilled tool plate.

## 4. Conclusions

High-quality protrusions are fabricated in the shape of elliptical and droplet cross-sections using Reverse-µEDM. The tool plate required for the Reverse-µEDM is fabricated using Nd: YAG-based LBµM at the optimized process parameters. The Nd: YAG LBµM parameters are analyzed to achieve burr-free, minimum taper, and shallow striation marks of micro-holes fabricated on a 0.5 mm thick titanium sheet. The micro-holes fabricated at optimal LBµM parameters are used as a tool plate in Reverse-µEDM for producing high-quality protrusions.

The following conclusions may be drawn upon the analysis of the fabricated products:

- The LBµM at the lowest duty cycle and current percentage, as the optimized LBμM parameters, resulted in minimum recast layer height, minimum taper, and average surface roughness (“*Ra_T_*”) with almost negligible burrs with shallow side wall striation marks of micro-holes.
- The pulse width of 0.25 ms, pulse frequency of 50 Hz, and a current percentage of 20% (% of avg. peak power) were the optimal parametric combinations for LBμM obtained by Grey relation analysis.
- The optimized LBμM parameters have demonstrated high-quality arrayed micro-holes and are further used to produce arrayed elliptical and droplet protrusions through Reverse-µEDM.
- Damage-free protrusions with an improved *MRR_P_*, *TWR*, and *Ra_P_* by more than 16%, 20% and 10%, respectively, are achieved by Reverse-µEDM upon using the optimized tool plate.

## Figures and Tables

**Table 1:** Factors and their levels.

Input Factors	Units	Level			
		1	2	3	4
Pulse width	ms	0.25	0.5	0.75	1
Pulse frequency	Hz	50	65	80	95
Current percentage	DC (%)	20	40	60	80

**Table 2:** Experimental runs and the recorded LBµM responses.

Factors					Responses				
	A	B	C	D = A × B × 10^−1^					
Exp No.	Pulse Width (ms)	Frequency (Hz)	Current (%)	Duty Cycle	*MRR_T_* (mm^3^/min)	Height of Recast Layer (mm)	*Ra_T_* (µm)	Taper (Rad)	Hv, HAZ (Micro-Holes)
**1**	**0.25**	**50**	**20**	**1.250**	**0.738**	**0.034**	**1.46**	**0.01**	**123**
2	0.25	65	40	1.625	0.753	0.042	1.78	0.029	128
3	0.25	80	60	2.000	0.782	0.049	1.91	0.043	130
4	0.25	95	80	2.375	0.807	0.056	2.02	0.051	134
5	0.50	50	40	2.500	0.831	0.062	2.14	0.068	139
6	0.50	65	20	3.250	0.878	0.078	2.58	0.077	145
7	0.50	80	80	4.000	0.911	0.083	2.87	0.112	151
8	0.50	95	60	4.750	0.963	0.107	3.17	0.124	162
9	0.75	50	60	3.750	0.876	0.071	2.47	0.094	156
10	0.75	65	80	4.875	0.942	0.093	2.98	0.132	163
11	0.75	80	20	6.000	1.015	0.111	3.58	0.159	171
12	0.75	95	40	7.125	1.062	0.123	3.95	0.181	179
13	1	50	80	5.000	0.952	0.096	3.02	0.146	165
14	1	65	60	6.500	1.029	0.119	3.70	0.168	173
15	1	80	40	8.000	1.094	0.128	4.35	0.211	183
16	1	95	20	9.500	1.108	0.132	4.68	0.237	188

**Table 3:** Process conditions of Reverse-μEDM and LBµM (Machine tool: Model: DT110i; Mikrotools Pte Ltd., Singapore).

Reverse-μEDM Parameters (Based on Expertise and Availability)		LBµM Parameters (Based on GRA Optimization)				
Setup	RC based	LASER type	Nd-YAG YLR-150/1500-QCW-MM-AC-Y11			
Resolution (X, Y, Z)	0.1 μm	Wavelength	1070 nm			
Tool plate	Titanium	Power	150 W			
Workpiece	Brass	Frequency	50 Hz			
Gap voltage	110 V	Pulse width	0.25 ms			
Capacitance	10 nF	Spot diameter	55 μm			
Electrode Feed rate	5 μm/s	current (%)	20			
Dielectric oil (type)	NICUT LL21 E					
**Measured Responses after Reverse-μEDM**						
**Reverse-μEDM** **Using** **Tool Plate Fabricated** **(Droplet Protrusions)**	**Approximately Time** **(h)**	***MRR_P_*** **(mm^3^/min)**	***TWR* (mm^3^/min)**	***Ra_P_*** (**μm)**	**Micro-Hardness of** **Tool Plate (Hv)**	
					**Before** **M/cing**	**After** **M/cing**
(I) with random parametric set	32	0.119	0.0053	1.63	118	125
(II) with optimal parametric set	28	0.142	0.0042	1.46	118	129

## Nomenclature

µEDM	Micro-electro-discharge-machining
Reverse-µEDM	Reverse-micro-electro-discharge-machining
LBµM	Laser beam micromachining
Nd: YAG	Neodymium-doped yttrium aluminum garnet
HAZ	Heat affected zone
RC	Resistance-capacitance
SEM	Scanning electron microscope
EDS	Energy Dispersive Spectroscopy
*TWR*	Tool wear rate (mm^3^/min)
GRA	Grey relational analysis
Hv	Vicker micro-hardness
*Ra_T_*	Average surface roughness of micro-holes side-wall surface (μm)
*Ra_P_*	Average surface roughness of protrusions surface (μm)
*MRR_T_*	Material removal rate (mm^3^/min) of tool plate
*MRR_P_*	Material removal rate (mm^3^/min) of protrusions
